# Reproductive seasonality, sex ratio and philopatry in Argentina's common vampire bats

**DOI:** 10.1098/rsos.160959

**Published:** 2017-04-26

**Authors:** H. A. Delpietro, R. G. Russo, G. G. Carter, R. D. Lord, G. L. Delpietro

**Affiliations:** 1 Servicio Nacional de Sanidad y Calidad Agroalimentaria (SENASA), Posadas, Argentina; 2 Smithsonian Tropical Research Institute, Panama, Republic of Panama; 3 Formerly of the Pan American Health Organization, Reading, PA, USA

**Keywords:** *Desmodus rotundus*, dispersal, rabies, reproductive seasonality, sex ratio, vampire bats

## Abstract

Common vampire bats (*Desmodus rotundus*) are a key rabies vector in South America. Improved management of this species requires long-term, region-specific information. To investigate patterns of demography and dispersal, we analysed 13 642 captures of common vampire bats in Northern Argentina from the period 1969–2004. In contrast with findings from more tropical regions, we found reproductive seasonality with peak pregnancy in September and peak lactation in February. Curiously, sex ratios were consistently male-biased both in maternity roosts and at foraging sites. Males comprised 57% of 9509 adults caught at night, 57% of 1078 juveniles caught at night, 57% of 603 juveniles caught in roosts during the day, and 55% of 103 newborns and mature fetuses. Most observed roosts were in man-made structures. Movements of 1.5–54 km were most frequent in adult males, followed by young males, adult females and young females. At night, males visited maternity roosts, and non-pregnant, non-lactating females visited bachelor roosts. Males fed earlier in the night. Finally, we report new longevity records for free-ranging vampire bats: 16 and 17 years of age for a female and male, respectively. Our results are consistent with model predictions that sex-biased movements might play a key role in rabies transmission between vampire bat populations.

## 1. Introduction

The common vampire bat (*Desmodus rotundus*) is an obligate blood-feeding bat found from northern Mexico to Uruguay and the centre of Chile and Argentina [1,2]. It is abundant in areas with livestock [3] and feeds mostly on the blood of domesticated cattle, horses, goats, pigs and sheep, and to a lesser extent—poultry, wildlife and humans [3–7]. As a primary reservoir of rabies in Latin America, vampire bats pose threats to wildlife, agriculture and human health. Vampire-bat-transmitted rabies outbreaks have killed thousands of livestock (annual damages estimated at $30 million USD) and dozens of people in a single year [8–12].

Previous studies have shown that vampire bats fit the typical mammalian pattern of female philopatry and male-biased dispersal [13–16]. Recent genetic analyses suggest that dispersing males play a key role in spreading the rabies virus between populations [14]. Viral expansions in Peru were seasonal and possibly linked to births and dispersal [14]. Models that forecast vampire-bat-transmitted rabies can therefore benefit from information on vampire bat demography, movement and reproduction. However, reports on these aspects of vampire bat ecology and behaviour can vary between different studies conducted across this species' wide geographical range [17]. Past long-term studies of social behaviour based on observations of individually marked vampire bats include 5 years of roost observations, genetic analyses and radio-tracking of marked bats in Costa Rica [15,16,18], and 3 years of mark–recaptures and serological and genetic analyses in Peru [12]. Here, we analysed 13 642 captures of common vampire bats and 2102 recaptures of marked bats in Northern Argentina from 1969 to 2004, to address reproductive seasonality, sex ratios, philopatry and dispersal. We asked the following main questions:

(1) *Is reproduction seasonal?* The majority of authors report an aseasonal polyestry for common vampire bats with births distributed randomly throughout the year [16,19–24], but other observations in Argentina, southern Brazil and Costa Rica suggest the existence of a spring peak of births [25–27].
(2) *Are sex ratios biased?* We examined evidence that sex ratios of both juvenile and adults were male-biased, both in maternity roosts and at foraging sites, and we discuss possible causes for this male-biased sex ratio.
(3) *How far and frequent are movements?* We examined evidence for movements between nearby roosts within a site (less than 1 km apart) and movements between more distant sites. The social structure of common vampire bats involves stable groups of females and their offspring and a few adult males in *maternity roosts*, and smaller groups of adult males or lone males in *bachelor roosts* [6,15,16,18,25,28,29]. Past studies found frequent roost-switching at sites in Mexico [27,30], Brazil [31] and Costa Rica [16,17], but not at other sites in Mexico [32] or Peru [12]. Wilkinson [15,16,18] observed that females maintained stable co-roosting associations while moving between different tree hollows, that a new female emigrated into female social networks about once every 2 years and that all young males dispersed from their natal group. In contrast with these observations of forest-dwelling vampire bats in Costa Rica, our observations of Argentinian vampires describe roosting behaviour in a diversity of roosts, many of which were frequently disturbed. These included man-made structures, such as bridges and abandoned buildings, natural caves and hollow trees.

## 2. Methods

### 2.1. Study site

**Figure 1.**
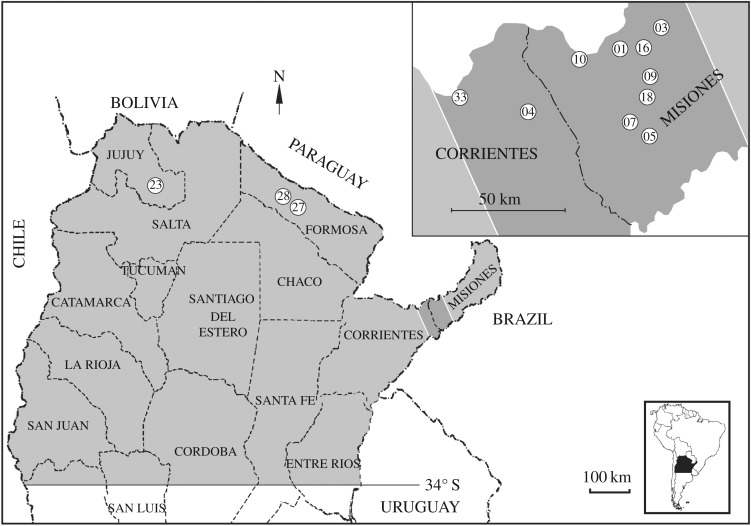
Study area. The approximate distribution of the common vampire bat (*Desmodus rotundus*) in Argentina is shown in light grey, and the most sampled area (dark grey) is shown in greater detail. White circles indicate the approximate location and identification number of the roosts mentioned in the text.

We conducted our study within the regional distribution of the common vampire bat in Argentina [33]. The climate in the northern region is warm and it is temperate in the central and southern regions, and rainfall is abundant in the eastern region (more than 1000 mm), which is without any dry season. In the central and western regions, the rains diminish (less than 1000 mm) and there is a dry winter season. Most of the native forest has been deforested for agriculture and ranching, and the native fauna has declined or disappeared in many places. Banding of vampire bats dates back to 1969, and our captures and observations occurred from 1973 to 2004 in the Argentine Provinces of Corrientes, Misiones, Entre Rios, Santa Fé, Chaco, Formosa, Jujuy, Salta, Tucumán, Santiago del Estero and Catamarca (figure 1).

### 2.2. Bat captures

We conducted 181 observations of roosts (electronic supplementary material, table S1) including two types of capture sessions: day roost captures and nocturnal captures (table 1). During day roost captures, vampires were caught inside roosts by a hand net, or in mist-nets as they tried to leave maternity roosts (43 capture sessions) or bachelor roosts (39 capture sessions).

**Table 1.** Captures (*N* = 13 642) of common vampire bats *Desmodus rotundus* in Argentina.

sample	*N* observed (banded)	age category	male	female	% male	95% CI % male
237 captures in mist-nets	10 587 (3491)	adult	5430	4079	57.1%	56–58%^a^
		juvenile	613	465	56.9%	54–60%^a^
43 captures in maternity roosts	2826 (0)	adult	688	1535	30.1%	29–33%
		juvenile	345	258	57.2%	53–61%^a^
38 captures in bachelor roosts	229 (0)	adult	203	26	88.6%	84–92%
		juvenile	0	0	—	—

^a^Indicates an expected 50/50 sex ratio.

To compare the numbers of males and females entering and exiting maternity roosts at night versus those remaining inside the same roosts by day, we conducted *successive roost captures*. First, we captured, sexed and counted bats in front of roosts with nets at night. Next, we captured, sexed and counted bats inside of the roosts after closing the roost entrances during the day. These paired captures could occur in either order and were separated by 24–48 h (table 2). We conducted paired successive captures twice in one roost and once in three other roosts (table 2).

**Table 2.** Successive captures of adults by sex in four maternal roosts at day and night.

roost (type)	coordinates	date	time	males/females	daytime change in % male, %female
23 El Tigre (cave)	24°01′10′′ S,	26 Oct 1984	day	10/23	−76%, −36%
	64°47′30′′ W	27 Oct 1984	night	42/36	
23 El Tigre		21 Mar 1985	day	9/16	−73%, +7%
		22 Mar 1985	night	33/15	
10 Garupá (building)	27°27′12′′S,	21 Jan 1991	night	20/13	
	55°48′42′′ W	23 Jan 1991	day	10/17	−50%, +31%
28 Bazán (tree)	24°27′20′′ S,	18 Aug 1993	night	12/14	
	60°50′06′′ W	20 Aug 1993	day	5/8	−58%, −43%
27 Lomitas (tree)	24°36′47′′ S,	21 Aug 1993	night	23/9	
	60°34′13′′ W	23 Aug 1993	day	10/18	−57%, +100%

During nocturnal captures, up to 20 mist-nets (typically 12 × 2.5 m) were placed at ground level. Nets were checked every 15–30 min and captured vampires were placed in individual bags. We conducted a total of 237 nocturnal captures, including 181 whole-night sessions, 39 sessions from dusk to midnight and 17 sessions from midnight to dawn. Some nocturnal captures were repeated at the same site at irregular intervals of 40 days to 14 years. On 142 of these capture nights, 3491 bats were banded on the forearm (left wing for males, right wing for females) with numbered metal bands (6.5 mm long, 3.5 mm diameter gape, Gey Band and Tag Company USA, and 7.5 mm long, 3.3 mm diameter gape, Mekaniska Sweden). Marked bats were released where they were caught. To assess the height of vampire flight, we recorded whether bats were entangled in the top or bottom half of the net on 31 capture nights.

### 2.3. Reproductive status

We tested for reproductive seasonality by observing the reproductive status and physical evidence of fighting in females and males across the year. Pregnancy in females was noted by abdominal palpation, massaging the abdomen with the thumb and index finger, which allows detection of fetuses that are 4–5 mm, including the vitelline and chorionic sacs [25]. Lactation was noted by squeezing the mammary glands and nipples.

Previous anecdotal observations suggested that testes size and aggression in males were greater in autumn (May) than during the rest of the year. To test this hypothesis, we calculated testicular circumference and counted recent wounds in males, as a measure of aggression. Observations were made in May and December of 2001 during nocturnal captures in front of roost #1 (Arroyo San Juan, located in the Department of Candelaria, Misiones Province, 27°26′11′′ S, 55°37′14′′ W—130 m AMSL, figure 1). Vampire testes are slightly elliptical, with a major cranial–caudal diameter (CD) and a minor transversal diameter (TD, electronic supplementary material 1, figure S1). Measurements were made with vernier callipers on the left testicle. We excluded monorchids, and those bats with a testicle that was less than 50% the size of the other testicle. The testicular circumference (TC) in mm was calculated as 3.14*(CD + TD)/2. We only counted wounds that were still bloody or recently scarred.

### 2.4. Age and sex

Juvenile bats were defined as those in which backlighting the finger joints revealed incomplete epiphyseal–diaphyseal fusion, showing an age less than nine months [25]. We assessed the sex ratios of adults, juvenile, newborns and fetuses. To evaluate the sex ratio of adults, we only used counts from nocturnal captures, because sex ratios from diurnal captures are biased by the proportion of known maternity and bachelor roosts. To evaluate the sex ratio of juveniles, we used counts from both nocturnal and diurnal captures, because juveniles only live in maternity roosts (table 1). We also measured sex ratios of captive-born newborns, defined as young bats with a piece of the umbilical cord still attached, and the sex ratios of mature unborn fetuses from wild and captive females. To calculate 95% confidence intervals for proportions, we used the prop.test function in R.

### 2.5. Roost observations

We observed 37 roosts. Many roosts were vandalized by people in an attempt to destroy the bats inside, and the majority were completely destroyed. For this reason, we could only analyse long-term recapture data from 12 surviving roosts (# 1, 3, 4, 5, 7, 9, 10, 16, 18, 33; figure 1) including three hollow trees, three man-made structures and six caves, that were vandalized but not completely destroyed. Due to the particular data-archiving methods we used, we could only analyse the total number of banded bats in each of these 12 roosts (electronic supplementary material 2), and the identity and ages of bats captured twice in the same roost (electronic supplementary material 3). We defined this non-random sample of individuals as *roost-faithful* bats. To test for a difference in frequency of roost-faithful bats of each sex across the 12 roosts, we fit a general linear model for predicting numbers of roost-faithful bats of each sex in a roost, with sex as a fixed effect, and roost and number of banded bats in that roost as random effects.

We measured *roost fidelity* of roost-faithful bats as the time between the first and last recapture at the same roost. For measuring time in days, we converted all years to 365 days and all months to 30 days. Wilkinson [15] noted that all year-old males that he observed (*n* = 17) dispersed between 12–18 months of age, while no females dispersed. We therefore defined ‘young’ bats as those first banded as volant juveniles (i.e. two to nine months old) and then later observed in the roost fewer than nine months later, meaning that they were less than 18 months old when first seen at the roost. All other bats, more than 18 months old, were considered ‘old’. To assess predictors of roost fidelity of roost-faithful bats, we fit a general linear mixed model with natural log-transformed roost fidelity as the response. Fixed factors were age category (young or old), roost type (tree, building, cave), sex (male, female), the interaction between sex and roost type, and the interaction between age and sex. Roost was a random factor nested within roost type.

### 2.6. Rabies samples

Samples of saliva, serum, salivary glands, brown fat, lung, and muscle of some live bats and all dead bats found inside or near roosts were sent to a laboratory for virological or serological testing (see [28,34–38]). We also tested samples from other wild animals found sick or dead during outbreaks of vampire bat rabies [39].

## 3. Results

### 3.1. Bat captures

Over 237 nights, 10 587 common vampire bats were caught in mist-nets, and 3489 of these bats were banded (table 1). Of these banded bats, 60% (2102) were later recaptured at least once, including 1262 bats recaptured only once, 527 bats recaptured twice, 216 bats recaptured three times, 59 bats recaptured four times, 28 bats recaptured five times, six bats recaptured six times, three bats recaptured seven times and one bat recaptured eight times, for a total of 3405 total recaptures. Males were both captured and recaptured more often (Fisher's exact test, *p* = 0.0001); we recaptured 55.6% of 1521 banded females and 63.8% of 1968 banded males.

### 3.2. Reproductive seasonality

**Figure 2.**
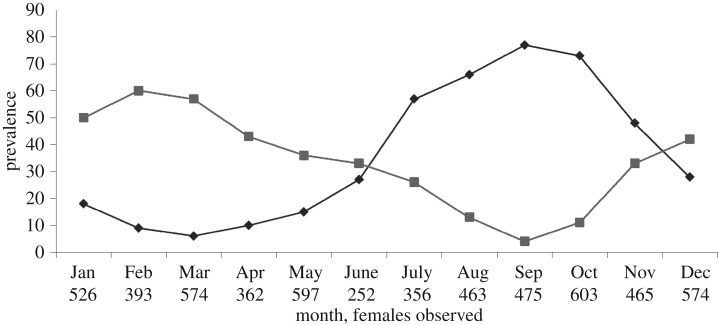
Reproductive seasonality. Prevalence (%) of adult females that were pregnant (diamonds) or lactating (squares). Numbers below month are sample sizes.

The 5640 adult female captures showed that monthly prevalence of pregnancy and lactation were negatively correlated throughout the year (Pearson's *r* = −0.874, *n* = 12, *p* = 0.0002). Pregnancy rates increased from 6% of females in March and peaked at 77% of females in September, while the presence of lactating females increased from 4% in September to a peak of 60% in February (figure 2). Mean testicular circumference was 21.6 mm in May (*n* = 75) and 19.8 mm in December (*n* = 68, *t* = −5.3, *p* < 0.0001), and the mean testicular circumference of the 25 recaptured bats decreased from 21.8 to 20.3 mm (paired-*t* *=* 4.3, *p* *=* 0.0003). Recent wounds were observed more often in May (9 of 75 bats) than in December (2 of 68 bats; *χ*^2^ = 4.475, *p* = 0.0344).

### 3.3. Age and reproduction

Relatively old bats were still reproductive. Two pregnant females were banded as adults more than 10 and 13 years previously, and three lactating females were banded 10 and 11 years previously. Three males with normal appearance and testes size were banded as adults 11, 12 and 13 years previously. The oldest vampires we observed were a male and female that were banded as adults on 29 April 1969 and 13 September 1969, and recaptured on, respectively, 14 June 1985 (at least 17 years old) and 13 December 1984 (at least 16 years old). To our knowledge, these are the highest age records for wild vampire bats. The physical appearance of the female was normal, and she was neither pregnant nor lactating. The male had small testicles, several old scars on the head and face, and had missing or very deteriorated canine and incisor teeth.

### 3.4. Sex ratios

We consistently observed male-biased sex ratios. Males comprised 57.1% of 9509 adults caught at night, 56.9% of 1078 juveniles caught at night and 57.2% of 603 juveniles caught in roosts during the day (table 1). The male-biased sex ratios in adults and juveniles were observed throughout the year (table 3). In newborns and mature fetuses, we observed 46 females and 57 males (table 4). This sample was not large enough to statistically demonstrate such a small bias (e.g. detecting that a 55% male sex ratio differs from chance with an 80% probability requires 776 observations); however, the observed 55% male sex ratio for newborns and fetuses was consistent with the estimated sex ratio in the larger sample of juveniles (95% CI: 53–61%, table 1).

**Table 3.** Male-biased sex ratios in nocturnal captures by month in Argentina.

month	adults	% male	juvenile	% male
Jan	1064	60	22	64
Feb	774	61	223	52
Mar	941	54	189	64
Apr	569	58	114	52
May	860	56	62	52
June	307	62	47	51
July	566	56	46	54
Aug	753	60	41	83
Sep	796	54	129	65
Oct	952	52	111	52
Nov	801	60	56	46
Dec	1126	57	38	61

**Table 4.** Sex ratios of mature fetus and newborn common vampire bats.

	newborn		mature fetus	
sample period	male	female	male	female
18 Sep 1971	—	—	12	10
May 1970–Oct 1971	3	4	3	1
Apr 1972–Apr 1976	19	12		
May 1992–Apr 1996^a^	7	9		
Mar 1997–Oct 2000	13	10		
total	42	35	15	11

^a^Data from Delpietro & Russo [25].

### 3.5. Bat movements

**Figure 3.**
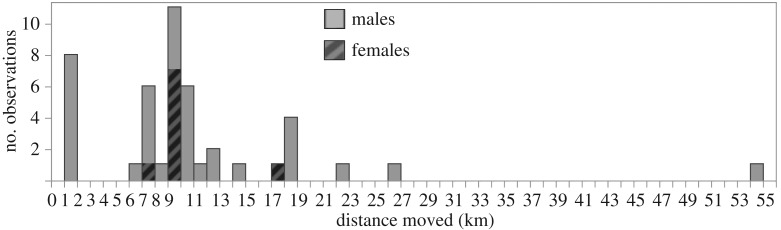
Observed movements by male and female common vampire bats.

Of the 2102 recaptured bats, we observed 45 cases of movements between roosts (figure 3, electronic supplementary material 4). These movements occurred more often in males than in females (*χ*^2^ = 7.7 d.f. = 1, *p* = 0.0057). The average observed distances travelled, however, were similar for males (median = 9.55 km) and females (median = 9.50 km). Of the 1256 recaptured males, we observed that 3% of them (36 bats) moved to roosts 1.5–54 km away. The remaining 97% of recaptured males remained within 1 km after 22–5671 days. Of the 837 recaptured females, we observed that 1% of them (9 bats) moved to roosts 8–18 km away. The remaining 99% of recaptured females were still within 1 km after 10–4491 days. The months between observations predicted the log-transformed distance travelled by females (*R*^2^ = 0.54, *p* = 0.024) and males (*R*^2^ = 0.14, *p* = 0.025), but we do not know the actual time used by the bats to travel those distances, and this correlation could be due to non-random sampling of sites.

The higher male dispersal rate was largely driven by juveniles, because the male bias was weaker when only inspecting adults (*χ*^2^ = 4.18, d.f. = 1, *p* = 0.041). Of the 365 juvenile males we banded, we later recaptured 51% of them (185 bats) within 1 km of their original capture site and we confirmed that 2% of them (8 bats) had moved to a farther site. Of the 277 juvenile females we banded, we recaptured 74% of them (206 bats) within 1 km of their capture site and we observed that 0.4% (just one bat) had moved to a farther site. Observed dispersal was thus higher for juvenile males than for juvenile females (*χ*^2^ = 6.09, d.f. = 1, *p* = 0.0136).

The lower recapture rates of juvenile males at the original site (*χ*^2^ = 8.88, d.f. = 1, *p* = 0.0029) was also unlikely to be caused solely by male-biased mortality because we detected no sex difference in the declining number of bats captured from one to six times (general linear model with natural log-transformed number of bats as response, and sex, times recaptured and their interaction as effects: *R*^2^ > 0.99, no interaction effect: *F*_1_ = 2.97, *p* = 0.13; male slope: −0.90, female slope: −0.96; electronic supplementary material, figure S2).

### 3.6. Roost selection

We observed 44 roosts in man-made structures, 19 in large hollow trees (more than 0.6 m^3^ hollow) of the species Ibirapitá (*Peltophorum dubium*), Timbó (*Enterolobium contortisiliquum*) and Samohú (*Chorisia speciosa*), and 18 in natural caves that ranged between roughly 1.5 and 50 m^3^ in size. The size of roost entrances ranged from 0.3 m^2^ to the 20–30 m^2^ entrance to a mine and the 30 m^2^ entrance to an abandoned warehouse with a collapsed wall. The height of entrances were 0–0.5 m for 64 roosts, 0.5–1.65 m for 14 roosts, 2.70 m for one roost and unknown for two other roosts.

A few other bat species were seen sharing the roosts with vampires. In a cave in the Province of Misiones, a group of vampires was seen hanging separately from a group of insectivorous *Macrophyllum macrophyllum* and another group of frugivorous *Carollia perspicillata*. On three occasions, we saw groups of 2 to 8 carnivorous *Chrotopterus auritus* roosting in the upper part of the mouth of caves that also housed vampires. In the Provinces of Salta and Catamarca we saw insectivorous bats (most probably Molossidae and/or Vespertilionidae) living in a different section of an abandoned mine.

### 3.7. Roosting behaviour

During diurnal captures, we captured and counted 2826 bats in 43 maternity roosts and 299 bats in 38 bachelor roosts (table 1). The size of maternity roosts ranged from 11 to 351 bats (mean = 66, median = 43, s.d. = 63.9). Bachelor roost size was smaller (Mann–Whitney test, *W* = 36, *p* < 0.0001) by an order of magnitude, ranging from 1 to 17 bats (mean = 6, median = 5, s.d. = 4). Females were occasionally observed inside of bachelor roosts (table 1) and these were all non-lactating adults without palpable pregnancy. During paired successive captures, more males were leaving or entering roosts by night than the number that remained inside of the same roosts by day (*t*_4_ = −3.67, *p* = 0.02; table 2). In contrast, we detected no difference in the number of females leaving or entering roosts at night and the number that remained inside by day (*t*_4_ = −0.26, *p* = 0.81; table 2).

### 3.8. Roost fidelity

**Figure 4.**
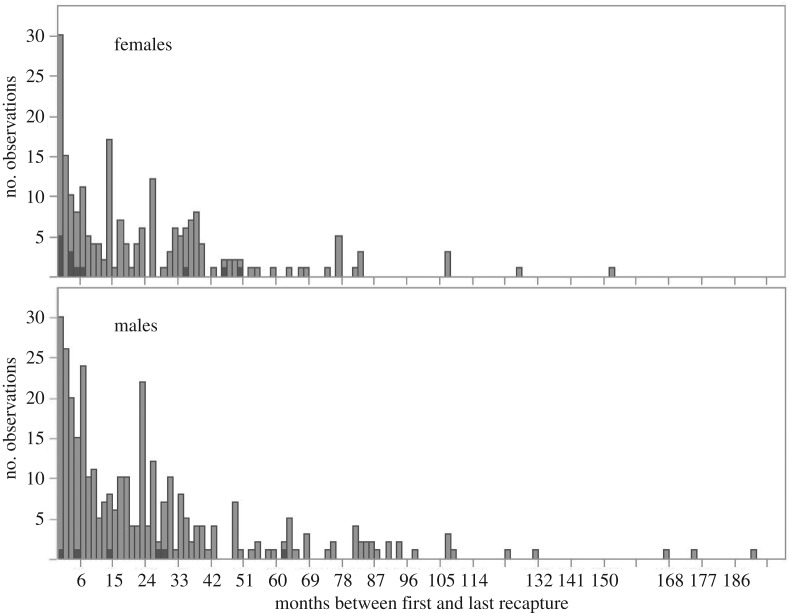
Maximum duration of roost use. Months between first and last recapture of bats captured twice in the same roost that were less than 1 year old when first seen in the roost (dark grey) or older (light grey).

Only 15% of banded bats were roost-faithful (electronic supplementary material 1, table S2), and the others had either died or switched roosts. The 539 roost-faithful bats were recaptured at the same roost 8–5751 days (15.8 years) later, with 57.5% of these recaptures being more than a year later (figure 4). Across the 12 observed surviving roosts, we did not detect any strong effects of sex (*F*_1,17_ = 0.26, *p* = 0.6), roost type (*F*_2,17_ = 0.9) or their interaction (*F*_2,17_ = 0.55, *p* = 0.6) on the number of roost-faithful bats in each roost (electronic supplementary material 1, table S2).

The duration of observed roost fidelity in roost-faithful bats was predicted by an interaction between roost type and sex (electronic supplementary material 1, table S3). When comparing the effect of sex separately within each roost type (with roost as a random factor), we found no clear effect of sex on roost fidelity in caves or hollow trees, but females showed greater roost fidelity in buildings (*F*_1,48.7_ = 6.4, *p* = 0.0146). However, this effect was largely driven by bats in roost 10, which suffered an atypically high rate of disturbance (see discussion).

Of the 539 roost-faithful bats, 3.5% (13 females and six males) were banded at foraging sites when juvenile and were thus less than a year old when first seen in the roost. Only 37% of these bats (three females and four males) were still in the same roost more than a year later (figure 4). In contrast, 93.5% (191 females and 313 males) of the roost-faithful bats were more than 18 months old when first seen in a roost, and 59% of these bats (119 females and 178 males) were seen again in the same roost more than a year later (figure 4).

### 3.9. Foraging behaviour

On 31 capture nights, 82% of 1864 bats were entangled in the bottom half of the net ($\chi_{1}^{2}=770$, *p* < 0.0001). We caught 370 males and 471 females that had fed before being captured. Of these fed bats, we observed that, from dusk to midnight, 64% were male (77 bats) and 36% were female (43 bats). From midnight to dawn, 41% were male (293 bats) and 59% were female (428 bats). These data suggest that males were more likely to feed earlier in the night ($\chi_{1}^{2}=23.1$, *p* < 0.0001).

### 3.10. Successive feeding in the same bite and males displaced by females

On several cases during June 1987, May 1989 and June 1991, at corrals of the Salta and Catamarca provinces, we observed two or three bats feeding successively on the same wound on the ears of goats and sheep. We saw two cases of female bats displacing males at feeding sites. On 6 February 1973, in the locality of San Carlos, province of Corrientes, in the forest of the Estancia Rincón Chico, approximately 300 m from a tree roost (#4, 27°38′50′′ S, 56°02′21′′ W), we observed, between 01.00 and 02.00, a male vampire feeding on the scapular area of a horse that was tied to a tree. Two to three minutes later, a female landed on the loin of the horse. A few seconds later, the male took flight and the female took over the abandoned bite and began feeding there.

On 17 May 1991, in a pig farm situated in the locality of Pomancillo, province of Catamarca (29°19′35′′ S, 65°43′00′′ W), between 01.50 and 02.05, we observed one scrotal male vampire bat feeding from a wound on the teat of a sleeping female pig. The bat had probably been feeding for at least two minutes because he was urinating. After less than one minute, an adult female bat, which may have been pregnant, landed on the rib–abdominal area of the sow about 30 cm from the first bat, and then began slowly approaching the male bat. Before they came into contact, the male bat took flight and the female bat began to feed on the abandoned bite.

## 4. Discussion

### 4.1. Reproductive seasonality

Our data confirm the hypothesis that vampire bat reproduction in Argentina shows a peak of births during the spring. Pregnancy and lactation rates were negatively correlated over time. At the end of winter (September), pregnancy rates reached a maximum and lactation reached a minimum. In March, the pregnancy rates diminished to a minimum and lactation rates peaked in February, illustrating a reproductive season with most births between the end of spring and early summer. This timing is consistent with mating occurring between the end of autumn and early winter given the gestation period of 165 and 180 days [25]. Larger testes sizes and higher aggression rates provide evidence of reproductive activity in males [40–42] and these measures are greater in May. Reproductive activity beyond these primary reproductive periods could be the consequence of secondary pregnancies after miscarriages or late pregnancies of young females with delayed sexual development [26].

The births of vampires in spring and summer coincide with the births of the majority of their domestic prey and also with their wild mammal prey in the central and southern part of South America [43,44]. Young animals might be easier for vampire bats to prey upon than older animals [45,46] because younger prey may bleed more and are less likely to have developed resistance to the anticoagulants of the bat saliva [47]. This reproductive seasonality, regardless of its origin, might therefore benefit the bats.

Although a female vampire may only produce a new offspring every year, individuals can live for more than 15 years in the wild [29,48] and more than 30 years in captivity (GG Carter 2016, unpublished data). We present new records of longevity for wild males (17+ years) and females (16+ years), and we observed males older than 11 and 12 years in good physical health and normal testicular size, and pregnant and lactating females older than 11 and 13 years.

### 4.2. Male-biased sex ratios

The group sizes and sex ratios of vampire bat maternity and bachelor roosts are consistent with previous reports in Argentina [6,25,28,29]. Sex ratios were consistently male-biased no matter whether juvenile or adults were sampled at nets, or juveniles were caught inside maternity roosts (table 1).

There is only mixed evidence for a biased sex ratio at birth in other datasets. During a study in Costa Rica, Wilkinson ([15,16,18]; GS Wilkinson 1985, unpublished data) did not detect a male bias in the sex ratios of every newborn bat he captured from roosts at La Pacifica (18 females and 15 males) or in the sample of infant or juveniles from roosts in Palo Verde (19 females and 23 males). On the other hand, all detected pregnancies in a captive population [49] led to the births of four females and 16 males (binomial test, *p* = 0.012). A second captive breeding colony in Panama produced five females and nine males (nine females versus 25 males: binomial test, *p* = 0.009).

The male bias we observed in Argentina was maintained throughout the year (table 3), with no obvious effects of sites differing in prey abundance [3], geography or ecology, as was reported in other bats [40,41,50]. It is unlikely that females were misidentified as males, given our experience with assessing sex in thousands of adults and juveniles, hundreds of newborns and dozens of mature fetuses. It is also unlikely that samples are biased solely by males being easier to catch in mist-nets, because this cannot explain the male bias of juveniles counted inside of the roosts after diurnal captures.

There are several possible adaptive explanations for biased sex ratios [50–53]. Although biased sex ratios have been studied for decades, the extent to which they are adaptive strategies remains controversial, especially in mammals [54], because biased sex ratios can be interpreted using a variety of post hoc explanations [55–58]. Trivers and Willard [53] argued that maternal condition should affect offspring fitness in mammals, and that this effect should often be stronger for male offspring than for female offspring; hence if males have more variable fitness and yield greater marginal fitness returns, then mothers in good condition should have male-biased offspring sex ratios. Trivers–Willard effects make the prediction that male-biased offspring are linked to better maternal condition, which might be assessed through prey availability. In a thriving population of vampire bats, all females may find themselves in better than average condition compared to the average ancestral conditions before the introduction of domesticated livestock to Latin America. This ‘miscalibration of maternal condition’ hypothesis predicts that females would produce more equal offspring sex ratios under conditions of lower prey availability.

On the other hand, several lines of reasoning could make the opposite prediction: male-biased sex ratios in vampire bats should be linked to low prey abundance. For instance, high prey density might bolster female fitness more than male fitness [59] or maternal effects could be strong enough to exceed the effects of sexual selection effects such that well-fed mothers are selected to produce more daughters [60]. A key problem with applying Trivers–Willard effects to our dataset is that vampire bat males are smaller than females, and the larger sex should be more costly for mothers to produce. Indeed, the relationship between male-biased sex ratios and maternal condition in other mammals is stronger when males are larger [61]. The male-biased sex ratio we observed therefore remains an intriguing mystery. Evaluating these adaptive hypotheses will require more and larger samples testing primary sex ratios.

### 4.3. Movements between sites

We recaptured the majority (60%) of bats that were banded, and almost all of these (97% of banded males and 99% of banded females) were recaptured within 1 km of the original site, from 10 days to 15.8 years later, suggesting that most bats are settled in a particular area. The 45 dispersal events (movements of more than 1 km from capture site) that we did observe were an unknown proportion of the actual dispersals that actually occurred, because we did not track bat movements and could not sample all possible roost sites. This sample of dispersal events, however, did show significant differences by age and sex. Males were more likely than females to move to far sites, and this difference was greater for younger bats. Relative observed dispersal rates were highest for adult males (3%, 28 of 891), followed by young males (2%, 8 of 365), adult females (1.5%, 8 of 560) and young females (0.4%, 1 of 206).

We found a few cases where young males appear to have remained at their natal site. Out of 365 banded juvenile males, we found four cases where a yearling male was observed at a roost and then seen there again more than a year later after the age of expected dispersal (12–18 months). It is important to note, however, the relative likelihood of observing events of dispersal versus non-dispersal. The chance of observing a surviving non-dispersing male is near 100%, but the chance of seeing a dispersing male is extremely low (the probability is the number of sampled roosts divided by the total possible roosts to which the bat could disperse). Despite this, we still observed twice as many dispersal events (*n* = 8) as non-dispersal events (*n* = 4) in males of dispersing age. Taken together, our capture and recapture data are therefore consistent with previous reports that many or most male and female adults remain in a given area, perhaps even for life, and that young males are several times more likely to disperse to new sites than young females [12,13,15,16,32,62].

### 4.4. Movements between roosts

Captures of non-pregnant, non-lactating adult females inside of bachelor roosts suggest that those females might visit those roosts for mating opportunities. Many more males were captured in front of the maternity roosts at night than were observed inside of the same roosts by day (table 2), corroborating previous reports that extra-harem males temporarily visit maternity roosts at night [15,16].

In addition to merely visiting other roosts at night, some movements suggested roost switching. We saw only a few of these roost-switching movements between the roosts we did observe, but only 15% of banded bats were seen again at the same roost. The remaining bats were either killed or had moved to unobserved roosts. Many of the bats seen repeatedly in the same roost used these roosts repeatedly for more than a decade.

We found no strong effects of sex, age or roost type on duration of observed roost fidelity, except that roost 10 showed greater roost fidelity among females. Roost 10 was an outlier for being the most disturbed and vandalized roost of the twelve that persisted long enough to be studied. Females, perhaps those with offspring, might be less likely than males to move from a roost when that roost is highly disturbed. We only observed complete roost abandonment on five occasions. In all cases, ranchers had moved the bats' cattle prey more than 1.8 km away. This suggests that proximity to livestock might influence vampire bat roost choice, as suggested by Turner [63].

Similar to past findings in Argentina [28], we found that more than half of the roosts were in barns, abandoned houses, wells, bridges and other man-made structures, illustrating that vampire bats can inhabit areas that lack natural roosts if prey are available. The entrances of vampire bat roosts tended to be large, which is likely to reduce risk of predation. However, we observed tracks of the crab-eating raccoon (*Procyon cancrivorus*) and grey-headed tayra (*Eira barbara*) inside or near roost entrances. We observed the savannah fox (*Cerdocyon thous*) inside roosts preying on fallen bats, and domestic cats catching vampire bats that were approaching goats, pigs, cattle or people. These observations demonstrate sources of predation risk for the bats as well as potential exposure of these predators to vampire-bat-transmitted rabies [39,64].

### 4.5. Sex differences in foraging times

Throughout the year, males fed earlier in the night. We speculate that females might tend to hunt later in the night than males because they might be more successful at displacing males from a wound than vice versa. Males might prefer to feed earlier to avoid females, which are larger, from taking over their bites, and/or to have more time to visit female roosts for mating opportunities (table 2).

### 4.6. The urine trail hypothesis

Roughly a quarter of the roosts in this study were found by landowners' dogs that appeared to follow odour trails on the ground. Vampire bats fly low to the ground [46,65], urination begins during feeding and can continue for more than 2 h [66–68], vampire bats possess a well-developed sense of smell [69,70] and the accumulation of many flights may create an odour trail that would allow for efficient commuting to prey. This opens the possibility that vampire bats leave urine-based odour trails on their repeated return flights.

### 4.7. Rabies

Rabies was the sole lethal disease we observed in vampire bats (see [28,34,36–38]). In Argentina vampire bat rabies outbreaks can appear at any time of the year and usually last less than 1 year in the same site, followed by an inter-epidemic period without rabies of 2 or more years [28,38]. During outbreaks, a large percentage (often more than 50%) of the local vampire bat population dies [28,35]. Rabid vampires typically lose their ability to fly, followed by paresis and paralysis and finally death in less than 48 hours after the onset of symptoms, but other vampire bats die without displaying symptoms. Rabies can increase aggression, which is conducive to virus transmission. Rabid vampire bats found on the floor of roosts often showed bites, evidence of aggression from conspecifics due to their atypical behaviour ([35]; electronic supplementary material 1, figure S3). Vampire bats showing rabid symptoms always died, and we never observed cases of recovery in those animals. After the outbreaks the virus was not found in surviving bats, though many of them carried rabies antibodies suggesting contacts with the rabies virus during the outbreak [34,37,38] and consistent with recent studies [12]. We also observed antibodies in non-vaccinated cattle that survived outbreaks of rabies (HA Delpietro, RD Lord, E Fuenzalida and JF Bell 1971, unpublished data), and in unvaccinated people who were bitten during an outbreak of vampire-transmitted rabies in Brazil (HA Delpietro, F Konolsaisen 1991, unpublished data; [71]). These observations confirm evidence that bats, cattle and people exposed to vampire-transmitted rabies virus can develop anti-rabies antibodies [12,71]. Our data are consistent with models of rabies transmission in which immunizing exposures, philopatry and dispersal play key roles in determining rabies transmission rates [8,12].

In summary, our recapture data are consistent with previous reports of female philopatry and dispersal of young males. Additionally, we found that, once settled, many adults of both sexes spend years and perhaps their lifetime at the same site. We also found several differences from reports of vampire bat reproduction and roosting ecology in other more Equatorial regions. We found that, despite frequent disturbance, vampire bats roosted primarily in man-made structures, reproduction was clearly seasonal, males foraged earlier in the night and sex ratios were male-biased for reasons that remain unclear.

## Supplementary Material

Additional tables and figures

## Supplementary Material

Roost data

## Supplementary Material

Recaptured bat data

## Supplementary Material

Dispersal events

## Data accessibility

Data for analyses are included in the electronic supplementary materials (electronic supplementary material 2 and 3) as .csv files.
