# Supplementary material for: Reproductive seasonality, sex ratio and philopatry in Argentina's common vampire bats
Source: R Soc Open Sci. 2017 Apr 26;4(4):160959. doi: 10.1098/rsos.160959 (PMC5414252; doi:10.1098/rsos.160959)
Supplement: Additional tables and figures [file rsos160959supp1.doc]

**Electronic supplementary material (ESM) 1**

Table S1. Sampling effort of the most studied roosts.

| Roost # | Roost observations /  observation period |
| --- | --- |
| 01 | 35 / 04-69 to 12-01 |
| 03 | 7 / 03-84 to 03-91 |
| 04 | 34 / 08-69 to 02-88 |
| 05 | 10 / 05-69 to 07-86 |
| 07 | 5 / 08-69 to 05-70 |
| 09 | 9 / 04-85 to 07-93 |
| 10 | 34 / 09-69 to 11-02 |
| 16 | 14 / 01-70 to 01-86 |
| 18 | 6 / 09-90 to 03-93 |
| 23 | 4 / 10-84 to 03-85 |
| 27 | 7 / 08-93 to 07-97 |
| 28 | 4 / 08-93 to 10-95 |
| 33 | 12 / 02-95 to 03-98 |

**Table S2. Proportion of roost-faithful bats in studied roosts.** Roost-faithful bats were those seen at least twice in the same roost.

| **Roost** | | **Banded** | | **Roost-faithful** | | **Proportion roost-faithful** | | |
| --- | --- | --- | --- | --- | --- | --- | --- | --- |
| ID | Type | male | female | Male | female | male | female | total |
| 1 | cave | 795 | 662 | 177 | 71 | 0.22 | 0.11 | 0.17 |
| 3 | cave | 72 | 52 | 8 | 10 | 0.11 | 0.19 | 0.15 |
| 4 | tree | 368 | 307 | 80 | 86 | 0.22 | 0.28 | 0.25 |
| 5 | cave | 118 | 78 | 3 | 4 | 0.03 | 0.05 | 0.04 |
| 7 | cave | 15 | 0 | 3 | 0 | 0.20 | -- | 0.20 |
| 9 | cave | 113 | 98 | 11 | 5 | 0.10 | 0.05 | 0.08 |
| 10 | building | 103 | 119 | 15 | 11 | 0.15 | 0.09 | 0.12 |
| 16 | building | 37 | 5 | 8 | 0 | 0.22 | 0 | 0.19 |
| 18 | cave | 112 | 124 | 12 | 5 | 0.11 | 0.04 | 0.07 |
| 27 | tree | 64 | 116 | 6 | 4 | 0.09 | 0.03 | 0.06 |
| 28 | tree | 17 | 17 | 1 | 0 | 0.06 | 0 | 0.03 |
| 33 | building | 63 | 65 | 4 | 15 | 0.06 | 0.23 | 0.15 |
| total |  | 1877 | 1643 | 328 | 211 | 0.17 | 0.13 | 0.15 |

**Table S3**. Predictors of roost fidelity in roost-faithful bats.

| **Fixed effect** | **DF** | **F** | **p** |
| --- | --- | --- | --- |
| Age | 1, 527.7 | 1.7 | 0.19 |
| Sex | 1, 530.8 | 0.03 | 0.87 |
| roost type | 2, 4.07 | 3.2 | 0.15 |
| age*sex | 1,522.2 | 3.1 | 0.08 |
| sex*roost type | 2, 333.6 | 4.1 | 0.018* |


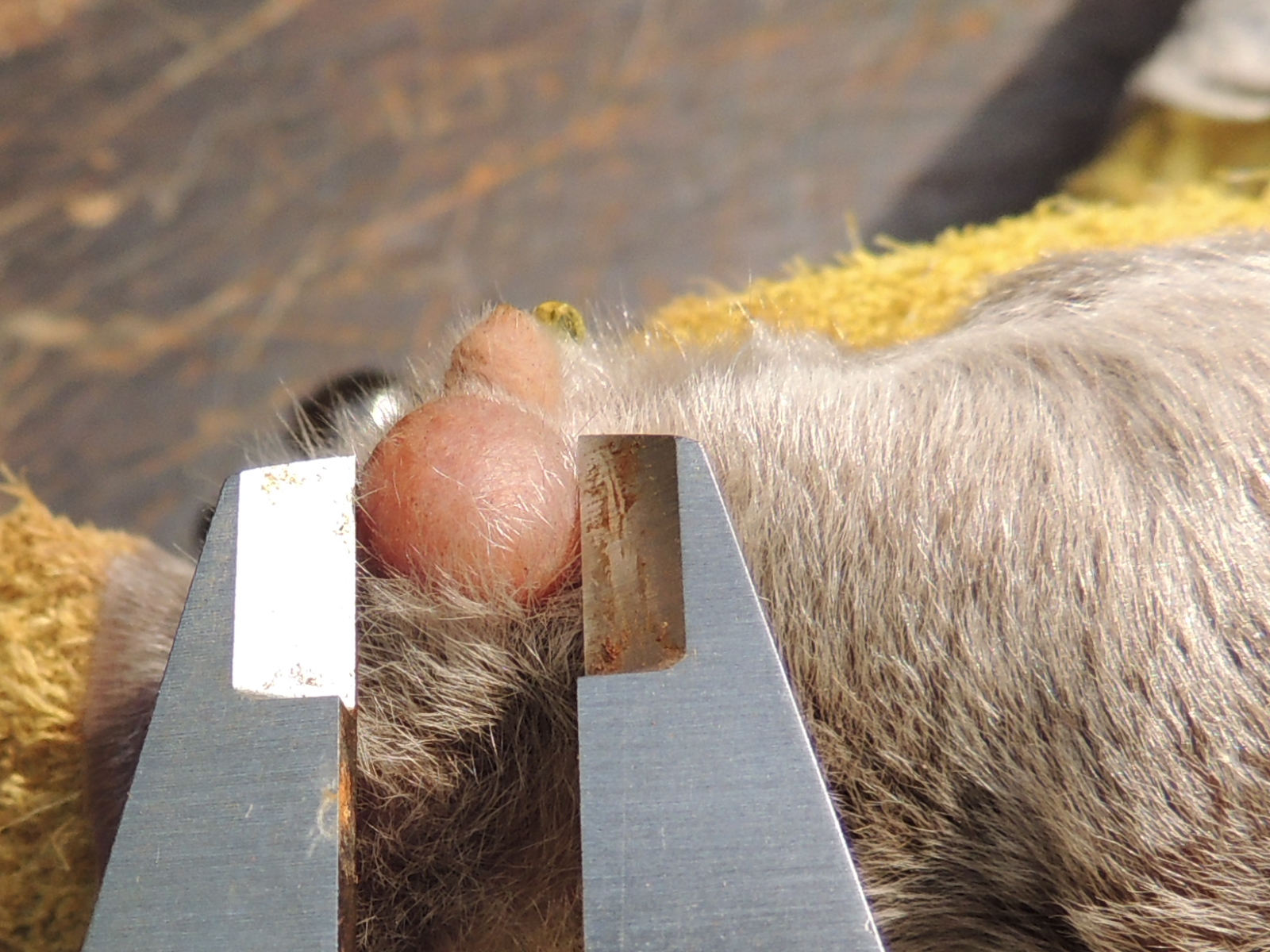


**Figure S1.** **Measurement of the craniocaudal diameter of the left testicle of a common vampire bat (*Desmodus rotundus*).** The form of the vampire bat testicle is slightly elliptical or oval, with a major diameter craniocaudal and a minor transversal.


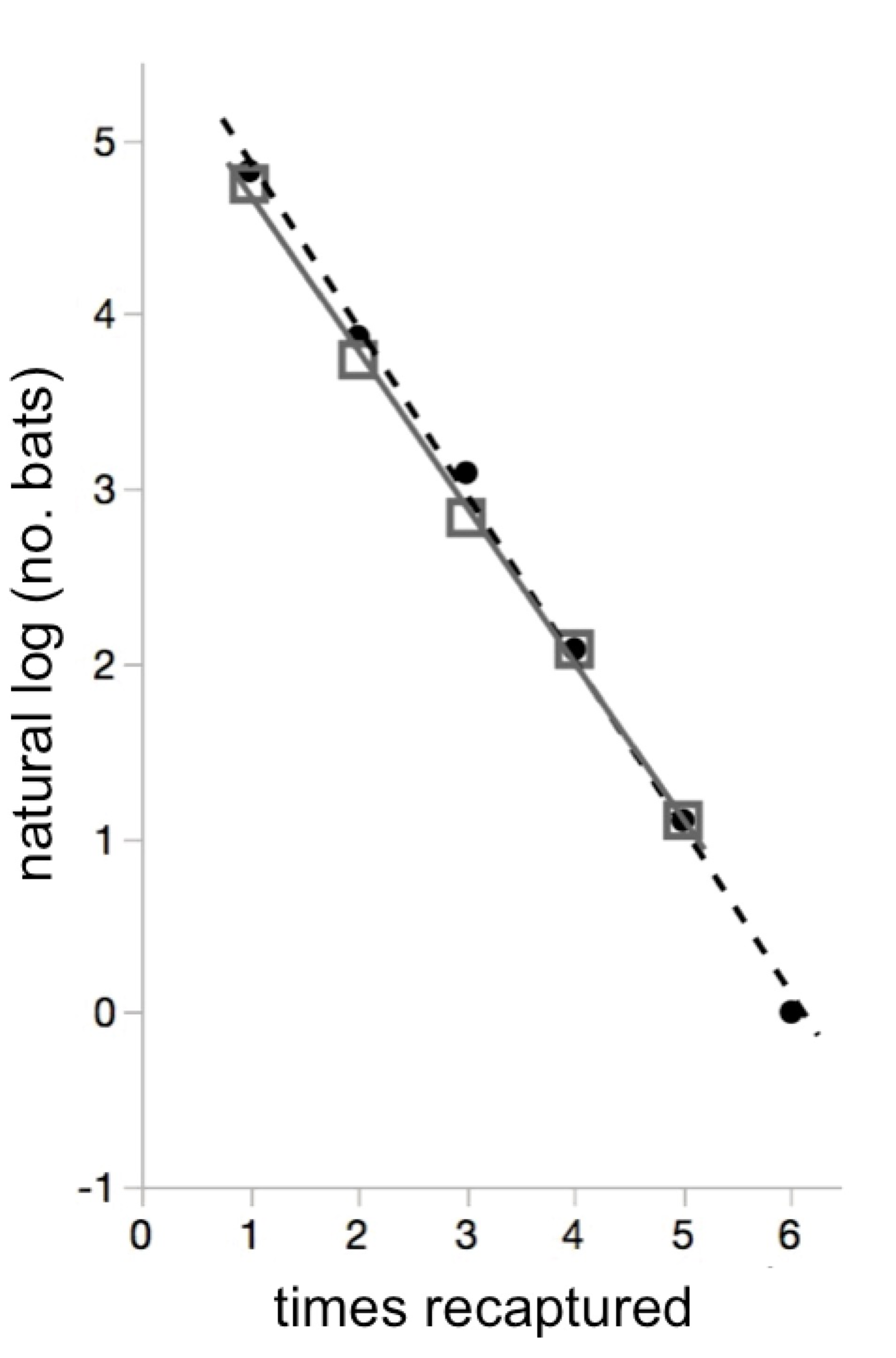


**Figure S2. No evidence for male-biased mortality driving recapture rates of each sex.** Plot shows slopes of natural log of number of bats caught from one to six times for females (dark circles, black dash lines) and males (open squares, gray solid line).


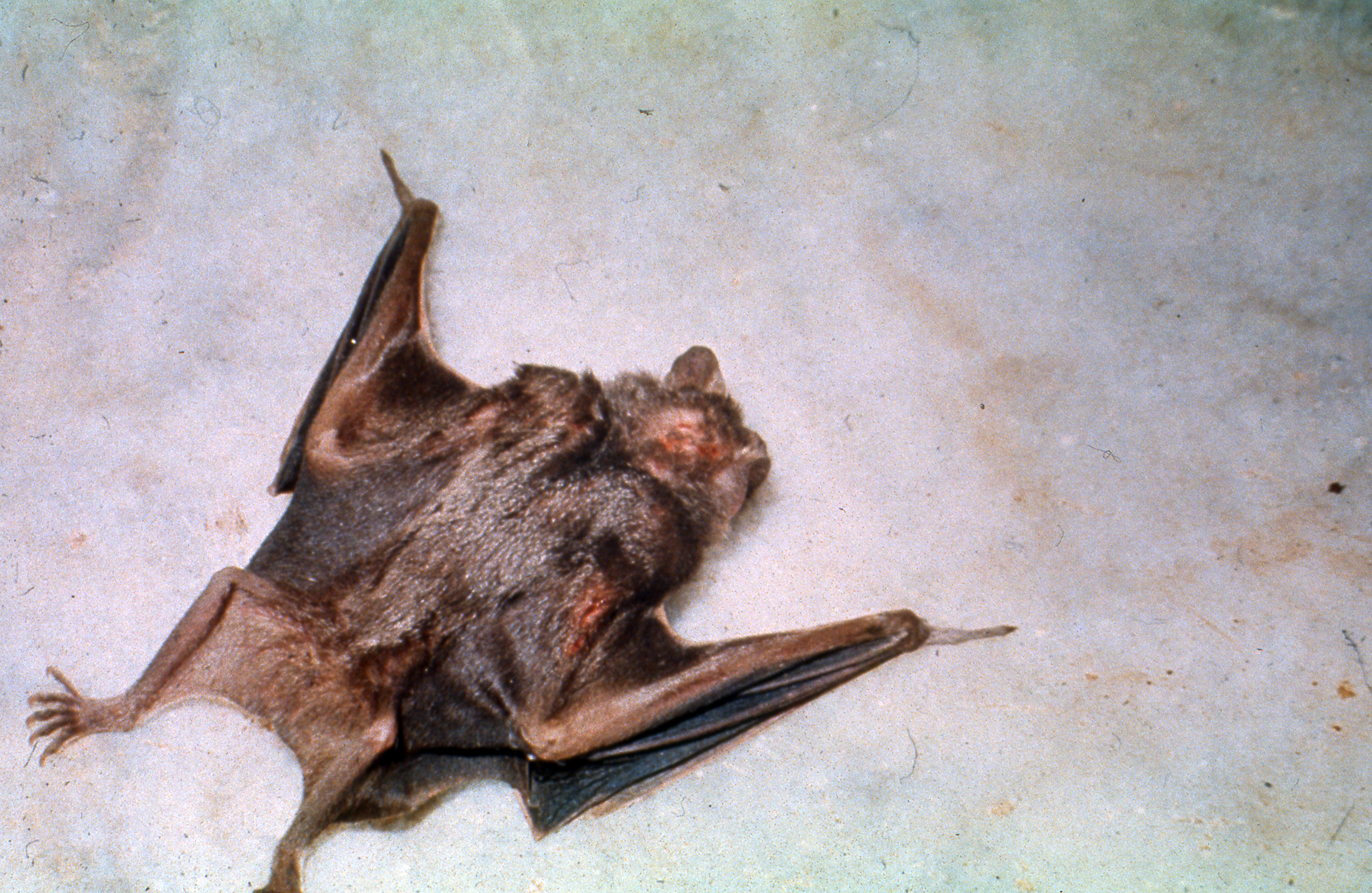


**Figure S3.** Rabid vampire bat showing typical bites of conspecifics on head, humerus, and uropatagium. The bat was paralyzed on the floor. Aggression to and from rabid bats is frequent and favours virus transmission. Rabid bats can maintain their biting ability until a few hours before death (Delpietro et al 1985).
